# Presenilin Promotes Dietary Copper Uptake

**DOI:** 10.1371/journal.pone.0062811

**Published:** 2013-05-07

**Authors:** Adam Southon, Mark A. Greenough, George Ganio, Ashley I. Bush, Richard Burke, James Camakaris

**Affiliations:** 1 Department of Genetics, The University of Melbourne, Victoria, Australia; 2 Florey Institute of Neuroscience and Mental Health, The University of Melbourne, Victoria, Australia; 3 School of Biological Sciences, Monash University, Victoria, Australia; Alexander Flemming Biomedical Sciences Research Center, Greece

## Abstract

Dietary copper is essential for multicellular organisms. Copper is redox active and required as a cofactor for enzymes such as the antioxidant Superoxide Dismutase 1 (SOD1). Copper dyshomeostasis has been implicated in Alzheimer’s disease. Mutations in the *presenilin* genes encoding PS1 and PS2 are major causes of early-onset familial Alzheimer’s disease. PS1 and PS2 are required for efficient copper uptake in mammalian systems. Here we demonstrate a conserved role for presenilin in dietary copper uptake in the fly *Drosophila melanogaster*. Ubiquitous RNA interference-mediated knockdown of the single *Drosophila presenilin* (*PSN*) gene is lethal. However, *PSN* knockdown in the midgut produces viable flies. These flies have reduced copper levels and are more tolerant to excess dietary copper. Expression of a copper-responsive EYFP construct was also lower in the midgut of these larvae, indicative of reduced dietary copper uptake. SOD activity was reduced by midgut *PSN* knockdown, and these flies were sensitive to the superoxide-inducing chemical paraquat. These data support presenilin being needed for dietary copper uptake in the gut and so impacting on SOD activity and tolerance to oxidative stress. These results are consistent with previous studies of mammalian presenilins, supporting a conserved role for these proteins in mediating copper uptake.

## Introduction

Presenilins are evolutionarily conserved proteins that function as the catalytic subunit of the γ-secretase multi-protein complex [1], [2]. γ-secretase functions in the intramembrane proteolysis of many substrates involved in diverse cellular processes including Notch, which has a well-characterized role in cell differentiation and development in diverse organisms including flies (*Drosophila melanogaster*) and mammals [2], [3]. The Alzheimer’s Precursor Protein (APP) is also cleaved by γ-secretase, and mutations in the two human presenilin genes encoding PS1 (*PSEN1*) and PS2 (*PSEN2*) are responsible for the majority of cases of early-onset familial Alzheimer’s disease (AD) [1], [2]. Recently our research groups demonstrated a novel role for PS1 and PS2 in regulating uptake of copper and zinc in mammalian systems [4].

Copper and zinc are essential micronutrients, and dyshomeostasis of these elements has been implicated in AD pathogenesis [5], [6]. Copper is a redox active metal required by numerous proteins including Superoxide Dismutase 1 (SOD1), an important cellular antioxidant [6], [7]. However, excess copper can be toxic through the production of reactive oxygen species that damage DNA, proteins and lipids [6], [7]. Zinc is structurally important for many proteins, including SOD1 [6], [8]. The uptake, sequestration, distribution and efflux of copper and zinc, therefore must be regulated at the cellular level and in the organism as a whole.

Mammalian dietary copper uptake occurs in the enterocytes of the gut where Copper Transporter 1 (CTR1) is required for apical copper uptake from the lumen and ATP7A, a P-type ATPase, is responsible for copper efflux across the basolateral membrane into the circulation [6], [7]. Copper transport in the midgut of the fly occurs similarly via the uptake proteins Ctr1A and Ctr1B and the efflux protein DmATP7 [9], [10]. Eukaryotic zinc homeostasis involves two families of transporters, the ZIP (SLC39) and ZnT (SLC30) proteins, and within enterocytes they are thought to contribute to apical zinc uptake and basolateral zinc export respectively [6], [8], [11]. The factors that regulate dietary uptake of copper and zinc in the gut of multicellular organisms remain to be fully elucidated.

Presenilin proteins are novel regulators of cellular copper and zinc uptake [4]. *PSEN1* heterozygous knockout mice have lower basal levels of copper and zinc in the brain and murine embryonic fibroblast (MEF) cells derived from *PSEN1* and *PSEN2* knockout mice have reduced uptake of copper and zinc [4]. SOD activity is also reduced in both *PSEN* knockout MEFs and brains of *PSEN1* heterozygous mice, consistent with a functional copper or zinc deficiency mediated by reduced presenilin activity [4]. We sought to utilise *Drosophila* to determine whether presenilin regulates dietary copper and zinc uptake. RNA interference (RNAi)-mediated knockdown of *Presenilin* (*PSN*), the single *Drosophila* orthologue, was associated with reduced copper bioavailability. Reduced *PSN* levels also impair SOD activity and the response to oxidative stress. We propose that presenilins have a conserved role in the regulation of dietary copper uptake.

## Materials and Methods

### Drosophila Stocks

All *Drosophila* strains were maintained on standard medium at 25°C. *w^1118^* (BL3605, Bloomington Stock Centre), *PSN* RNAi (KK101379, Vienna Drosophila RNAi Center), *pUAST*-*DmATP7* [12], MEX-GAL4 [13], HikoneR-GAL4 (gift from P. Daborn, University of Melbourne, Australia) [14], MTN-EYFP [15] (gift from W. Schaffner, University of Zurich, Switzerland), *w*; *IF*/*CyO*; *MKRS*/*TM6b*, (gift from G. Hime, University of Melbourne, Australia). *Drosophila* media was supplemented with additional copper (CuSO_4_, Sigma) or zinc (ZnSO_4_, Sigma) at concentrations indicated in figure legends. 0.5 mM bathocuproine disulfonic acid (BCS; Sigma) and 0.1 mM N,N,N,N-tetrakis(2pyridylmethyl)ethylenediamine (TPEN, Sigma) were added to media to limit available copper and zinc respectively. 10 mM paraquat (Sigma) was used to induce superoxide.

### Gene Expression

Quantitative polymerase chain reaction (Q-PCR) analysis of gene expression was used to confirm RNAi-mediated knockdown of *PSN* in replicates of 10 third instar larvae. RNA extraction, cDNA synthesis and Q-PCR were conducted as previously reported [16]. Forward and reverse *PSN* primer sequences were CTCGGTATTAGTGGGCAAGG and AAATGGCCAGAAGCAGAAGA, respectively. *RPL11* was used as a housekeeping gene [14].

### *Drosophila* Survival Assays

Larval survival to adulthood was determined as reported previously [17]. Replicates of 50 first instar larvae were scored for survival to adulthood on basal media and media supplemented with copper, BCS, zinc, TPEN or paraquat.

### Metal Analysis with Inductively Coupled Plasma Mass Spectrometry (ICP-MS)

*Drosophila* were reared until pupae on basal media and media supplemented with copper, BCS, zinc, or TPEN. Samples were digested in 65% nitric acid and copper, zinc, iron, and manganese levels were measured using an Agilent 7700 ICP-MS instrument (Agilent) as described previously [18].

### SDS-PAGE and Immunoblotting

*Drosophila* samples were lysed with Triton X-100 (Sigma) and protein was resolved on NuPAGE 4–12% Bis–Tris gels (Invitrogen) and transferred to nitrocellulose membranes for western immunoblotting. Primary antibodies used were rabbit anti-SOD1 (1∶250, FL-154 Santa Cruz), mouse anti-GFP (1∶1000, Roche) and mouse anti-α-Tubulin (1∶10000, Sigma). Horseradish peroxidase coupled secondary antibodies were rabbit anti-mouse and goat anti-rabbit (1∶7000, Dako). Chemiluminescence was detected using ECL (GE Healthcare) and images were captured with a Fujifilm LAS-3000 (Fujifilm LifeScience).

### Microscopy

An MTN-EYFP reporter construct was used as a proxy measure of larval copper distribution as previously described [12], [15]. Gut tissue from third instar larvae was dissected in cold PBS and immediately imaged with an Olympus SZX12 dissecting microscope using DP controller software (Olympus). MTN-EYFP expression was observed in the dissected midgut of 40 control (20 basal and 20 copper treated) and 40 *PSN* knockdown (20 basal and 20 copper treated) larvae over 5 repeat experiments. To allow direct comparisons between control and *PSN* knockdown larvae, images were captured from of a pair of larvae in a single field of view.

### SOD Activity

*Drosophila* third instar larvae were lysed with Triton X-100 (Sigma). SOD activity was measured with 4 µg of protein using a SOD assay kit (Dojindo) according to the manufacturer’s instructions and described previously [19]. Absorbance was read at 440 nm using a PowerWave XS microplate spectrophotometer (BioTek Instruments).

### Statistics

Statistical analyses were conducted with Prism 4 (GraphPad Software) as described in figure legends. Quantitative data is presented as the mean with S.E.M. and P<0.05 was deemed statistically significant.

## Results

The well-established UAS-GAL4 system was used for RNAi-mediated knockdown of *PSN* expression to determine whether presenilin contributes to metal homeostasis in *Drosophila*. Ubiquitous knockdown of *PSN* with the actin-GAL4 driver was lethal, consistent with the established role of PSN in developmental processes. However, viable flies were produced when *PSN* was knocked down specifically in tissues of the digestive system using the HikoneR or MEX GAL4 drivers. HikoneR-GAL4 drives expression in the midgut, fat body and Malpighian tubules, while MEX-GAL4 drives expression in the midgut only. Q-PCR analysis of whole third instar larvae confirmed HikoneR-GAL4 reduced *PSN* expression by 31±7%, relative to control. MEX-GAL4 reduced *PSN* expression by 34±8%, relative to control larvae. The midgut-specific MEX-GAL4 driver was used for subsequent studies to assess the role of PSN in dietary copper and zinc homeostasis.

**Figure 1:**
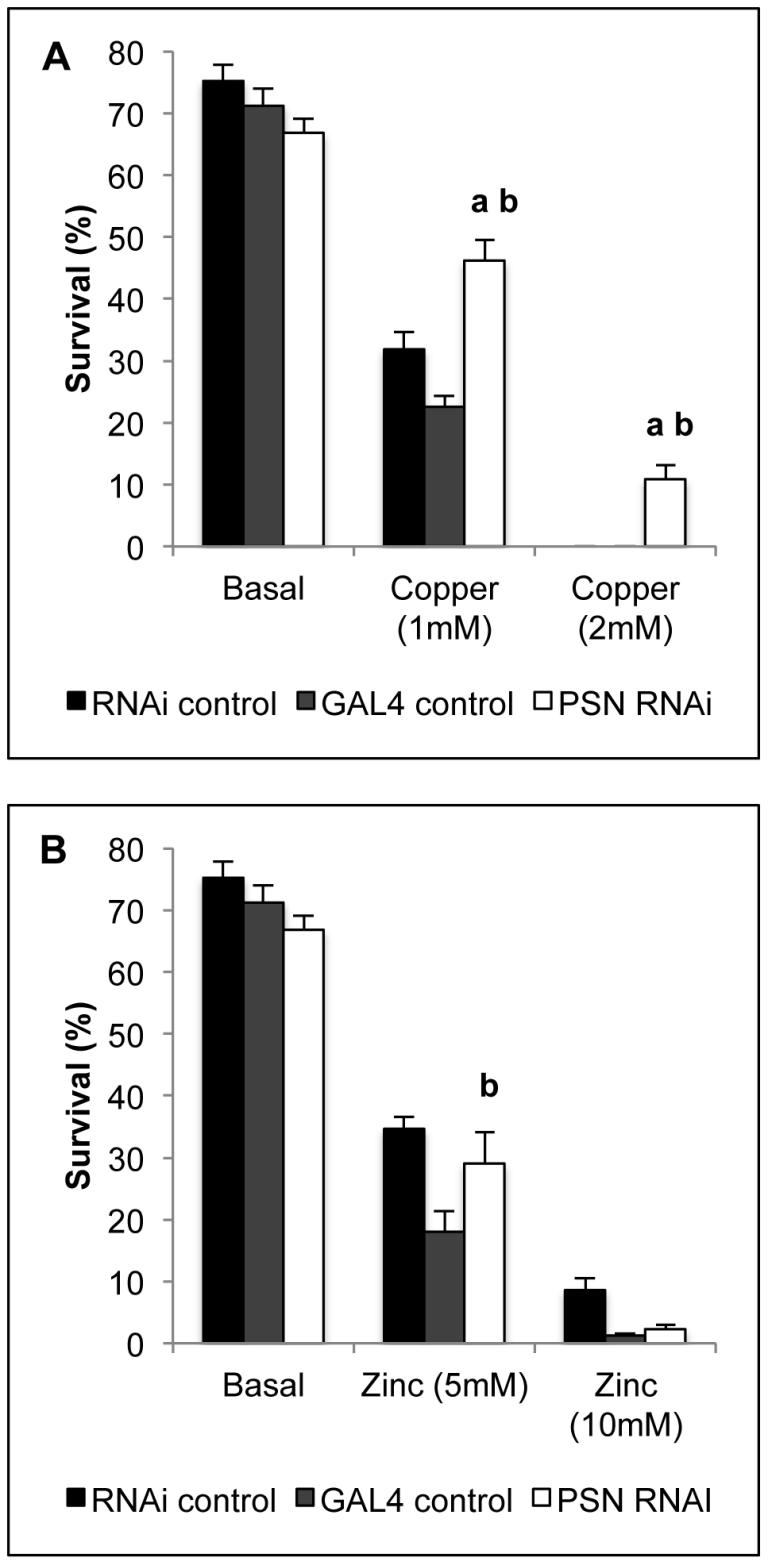
Metal tolerance in *Drosophila*. Copper tolerance (A) and zinc tolerance (B) were assessed by survival to adulthood on basal media and metal-supplemented media for control (RNAi control, GAL4 control) and midgut *PSN* knockdown (*PSN* RNAi) *Drosophila*. Values are percentage survival with S.E.M. from fifteen replicates of 50 *Drosophila*. *PSN* knockdown *Drosophila* were highly tolerant to copper-supplemented media. ^a^Significant difference from RNAi control, ^b^significant difference from GAL4 control, as determined by two-way ANOVA with Tukey’s post-hoc test (P<0.05).

Metal tolerance was examined in *Drosophila* reared on basal media and media supplemented with additional copper (Figure 1A) and zinc (Figure 1B). *w^1118^* control flies were crossed to both the PSN-RNAi stock and the MEX-GAL4 stock to create two control strains referred to as RNAi control and GAL4 control, respectively. Midgut *PSN* knockdown did not alter survival on basal media relative to either control strain, indicating the reduction in *PSN* levels did not have a major fitness cost. Survival on copper-supplemented media was much greater in the *PSN* knockdown flies (Figure 1A). This was significant relative to both controls and only *PSN* knockdown flies survived the highest copper concentration. Rearing *PSN* knockdown flies on media supplemented with the copper-chelator BCS did not significantly affect their survival (Figure S1). Survival on zinc-supplemented media was not consistently different between midgut *PSN* knockdown flies and controls (Figure 1B).

**Figure 2:**
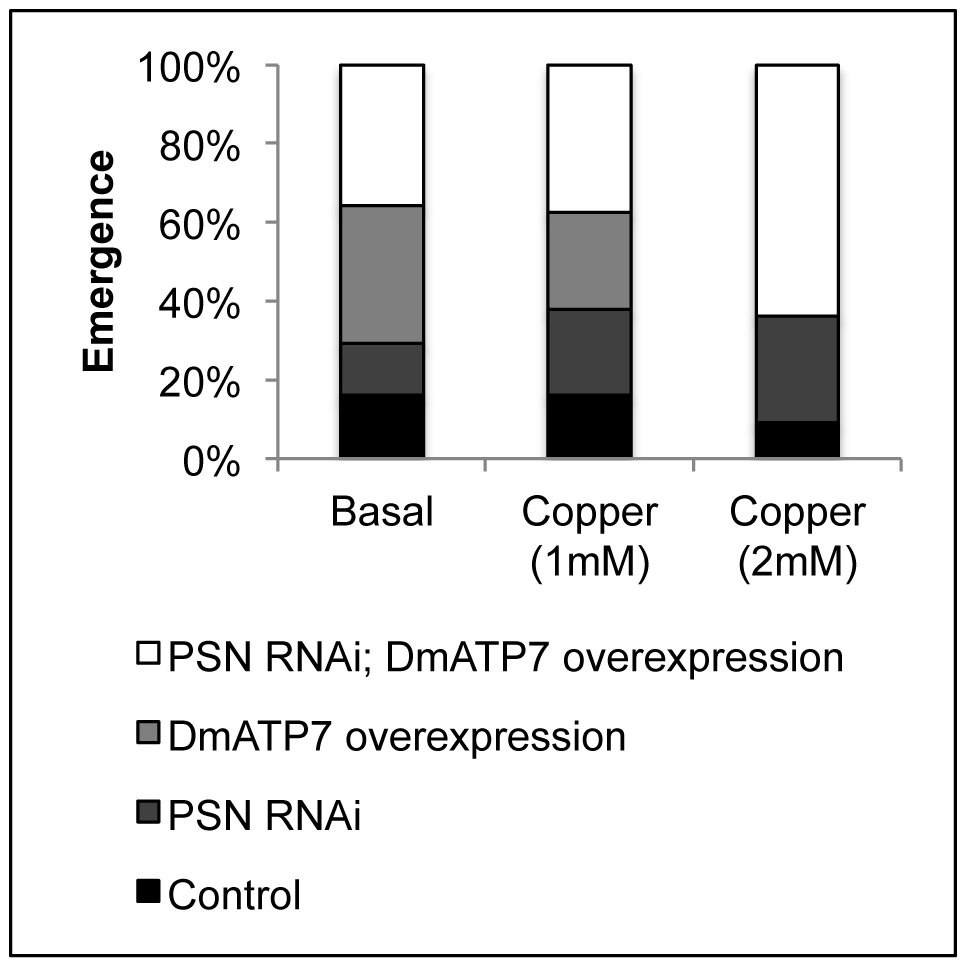
Copper tolerance in *Drosophila* overexpressing *DmATP7*. MEX-GAL4 was used to knockdown *PSN* in the midgut together with overexpression of the copper transporter *DmATP7*. Flies were allowed to lay eggs on basal media and copper-supplemented media and the genotype of the surviving offspring was determined: control (MEX-GAL4/*Cyo*; +/*TM6b*), *PSN* RNAi (MEX-GAL4/*PSN* RNAi; *TM6b*/+), *DmATP7* overexpression (MEX-GAL4/*Cyo*; *pUAST*-*DmATP7*/*TM6b*), *PSN* knockdown and *DmATP7* overexpression (MEX-GAL4/*PSN* RNAi; *pUAST*-*DmATP7*/*TM6b*). Emergence of each genotype was expressed as a proportion of the total number of adults that emerged on each media. Flies overexpressing *DmATP7* did not survive on 2 mM copper, but this copper-sensitivity was overcome by simultaneous knockdown of *PSN*.

We next sought to determine whether *PSN* knockdown could overcome the copper sensitivity caused by overexpression of *DmATP7* in the midgut. A compound heterozygous strain (*PSN*-RNAi/*Cyo*; *pUAST*-*DmATP7*/*TM6b*) was crossed with the homozygous MEX-GAL4 driver strain and allowed to lay eggs on basal media and media supplemented with additional copper (Figure 2). The emergence of each of the four genotypes was expressed as a proportion of the total number of adults that emerged on each media. Flies overexpressing *DmATP7* did not survive on 2 mM copper, but this copper-sensitivity was overcome by simultaneous knockdown of *PSN*. This is consistent with *PSN* knockdown affecting midgut copper homeostasis prior to copper export across the basolateral membrane into the circulation.

**Figure 3:**
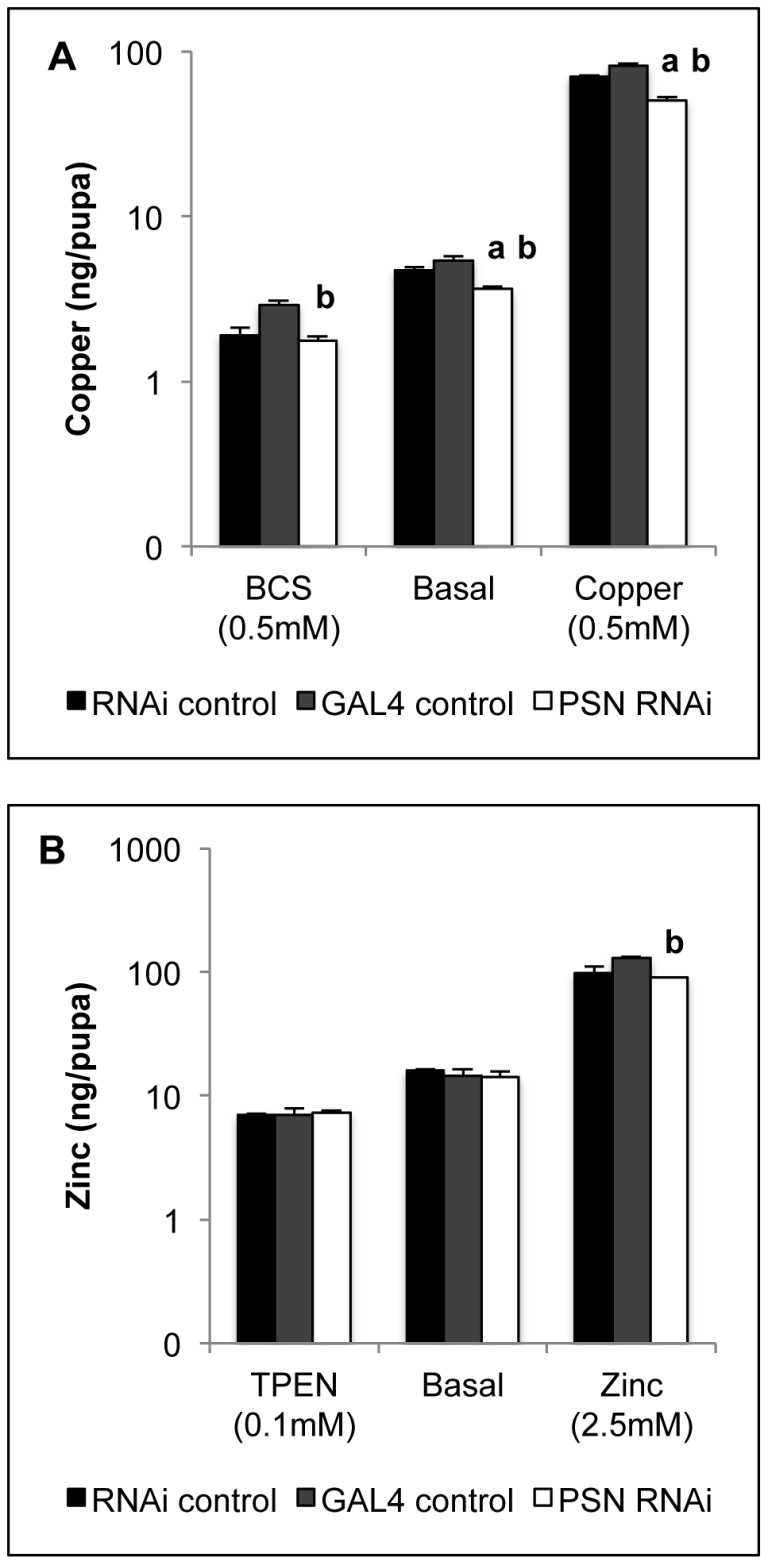
Metal accumulation in *Drosophila*. Copper (A) and zinc (B) levels were measured by ICP-MS in control (RNAi control, GAL4 control) and midgut *PSN* knockdown (PSN RNAi) pupae. *Drosophila* were reared on either copper chelator (0.5 mM BCS), basal media or 0.5 mM copper (A) or zinc chelator (0.1 mM TPEN), basal media or 2.5 mM zinc (B). Values are mean metal content per pupa with S.E.M. from five replicates of 20 pupae and shown on a logarithmic scale. *PSN* knockdown *Drosophila* accumulated less copper on basal media and copper-supplemented media. ^a^Significant difference from RNAi control, ^b^significant difference from GAL4 control, as determined by two-way ANOVA with Tukey’s post-hoc test (P<0.001).

We next measured metal levels to determine whether the copper tolerance seen with midgut *PSN* knockdown was caused by reduced dietary copper uptake. ICP-MS was used to measure metal levels in pupae, as this represents the end of the larval feeding stage (Figure 3, Figure S2). The weight of the *PSN* knockdown pupae (0.88±0.02 mg) was not significantly different to the RNAi control (0.84±0.02 mg) or GAL4 control (0.91±0.02 mg) pupae. Copper levels were measured in *Drosophila* reared on the copper-chelator BCS, basal media and also copper-supplemented media (Figure 3A). Zinc levels were measured in *Drosophila* reared on the zinc chelator TPEN, basal media and also zinc-supplemented media (Figure 3B). Relative to each control strain, midgut *PSN* knockdown was associated with significantly reduced copper levels when reared on copper-supplemented media and there was also a trend for lower levels on basal media (Figure 3A). Zinc levels were not consistently different between midgut *PSN* knockdown flies and controls when reared on zinc-limiting, basal or zinc-supplemented conditions (Figure 3B). *PSN* knockdown did not consistently alter basal levels of iron, manganese, calcium or magnesium (Figure S2).

**Figure 4:**
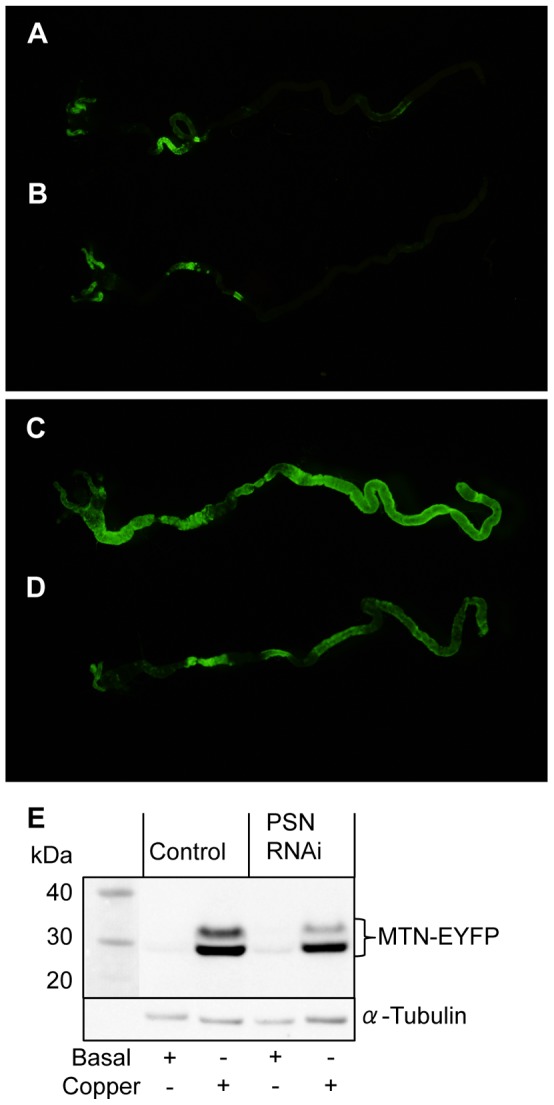
MTN-EYFP expression in *Drosophila*. *Drosophila* expressing a copper-inducible MTN-EYFP construct (green) were reared until third instar on basal media (A, B) and media supplemented with 0.5 mM copper (C, D). Gut tissue incorporating the gastric caecum and anterior midgut (left) to the posterior midgut (right) was dissected from control (A, C) and midgut *PSN* knockdown (B, D) larvae. MTN-EYFP expression was greater in the gut of control larvae reared on copper-supplemented media(C) than those on basal media (A), indicative of increased copper levels. Under basal condition, MTN-EYFP expression in the gut of PSN knockdown (B) and control (A) larvae was comparable. When reared on copper-supplemented media MTN-EYFP levels were lower in the gut of *PSN* knockdown larvae (D) than control (C), consistent with reduced copper levels. Western immunoblotting with a GFP antibody was used to confirm MTN-EYFP levels in pooled samples of five larvae reared on basal media and media supplemented with 0.5 mM copper (E). α-Tubulin was used as a loading control. When reared on copper-supplemented media MTN-EYFP levels were lower in the gut of *PSN* knockdown larvae than control.

Copper distribution within *Drosophila* larvae was assessed using flies expressing an EYFP construct containing a copper-responsive metallothionein promoter element (Figure 4). Control (Figure 4A, C) and midgut *PSN* knockdown (Figure 4B, D) *Drosophila* were reared until third instar on either basal media (Figure 4A, B) or media supplemented with 0.5 mM copper (Figure 4C, D). The midgut was dissected to include the gastric caecum and proventriculus (left) and the entire midgut terminating at the junction with the hindgut (right). When reared on basal media, EYFP expression was comparable between control (Figure 4A) and midgut *PSN* knockdown (Figure 4B) larvae, with strongest expression detected in the proventriculus and copper cell region of the midgut. When reared on copper-supplemented media, EYFP expression was markedly increased throughout the entire midgut of control larvae (Figure 4C), consistent with increased copper uptake relative to basal conditions (Figure 4A). EYFP expression was also increased in the midgut of *PSN* knockdown larvae reared on copper-supplemented media (Figure 4D), relative to those raised on basal media (Figure 4B). However, this increase in EYFP expression was not as large in the *PSN* knockdown larvae (Figure 4D) when compared to control (Figure 4C). Analysis of MTN-EYFP levels in third instar larvae, by western immunoblotting with an anti-GFP antibody, confirmed that copper-induced expression of MTN-EYFP was reduced by midgut *PSN* knockdown (Figure 4E). These results are indicative of reduced dietary copper uptake into the midgut of *PSN* knockdown larvae, which is consistent with their overall lower copper levels (Figure 3A) and increased copper tolerance (Figure 1A) when reared on copper-supplemented media.

**Figure 5:**
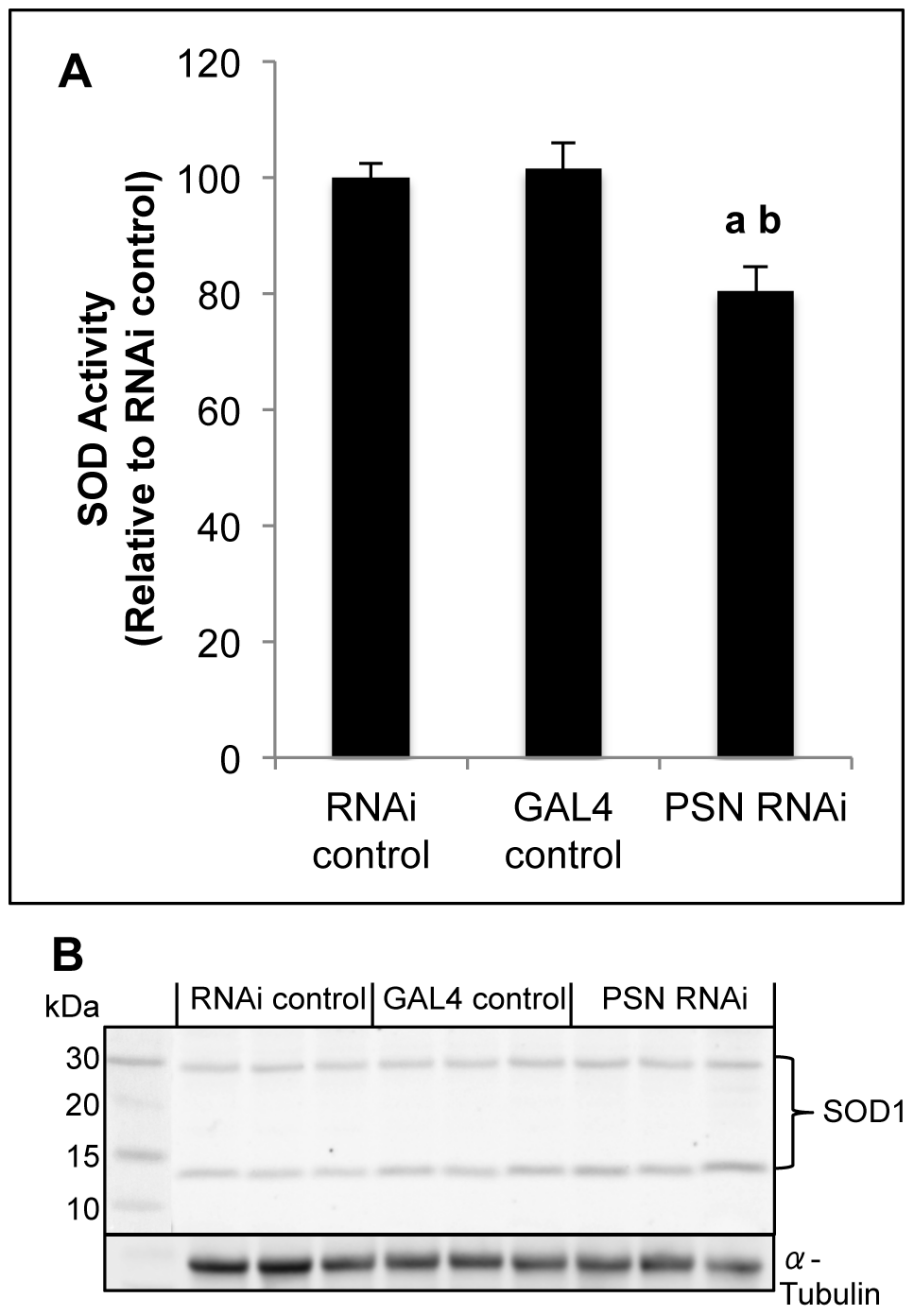
SOD activity in *Drosophila*. SOD activity (A) and SOD1 protein levels (B) were assessed for control (RNAi control, GAL4 control) and midgut *PSN* knockdown (PSN RNAi) third instar larvae. (A) SOD activity was measured colorimetrically and expressed relative to activity in RNAi control larvae. Values are mean activity with S.E.M. from twenty replicates of 10 larvae. *PSN* knockdown *Drosophila* had reduced SOD activity. ^a^Significant difference from RNAi control, ^b^significant difference from GAL4 control, as determined by one-way ANOVA with Tukey’s post-hoc test (P<0.05). (B) SOD1 monomer and dimer were identified by western immunoblotting with a SOD1 antibody. α-Tubulin used as a loading control. SOD1 protein levels were not reduced by *PSN* knockdown.

**Figure 6:**
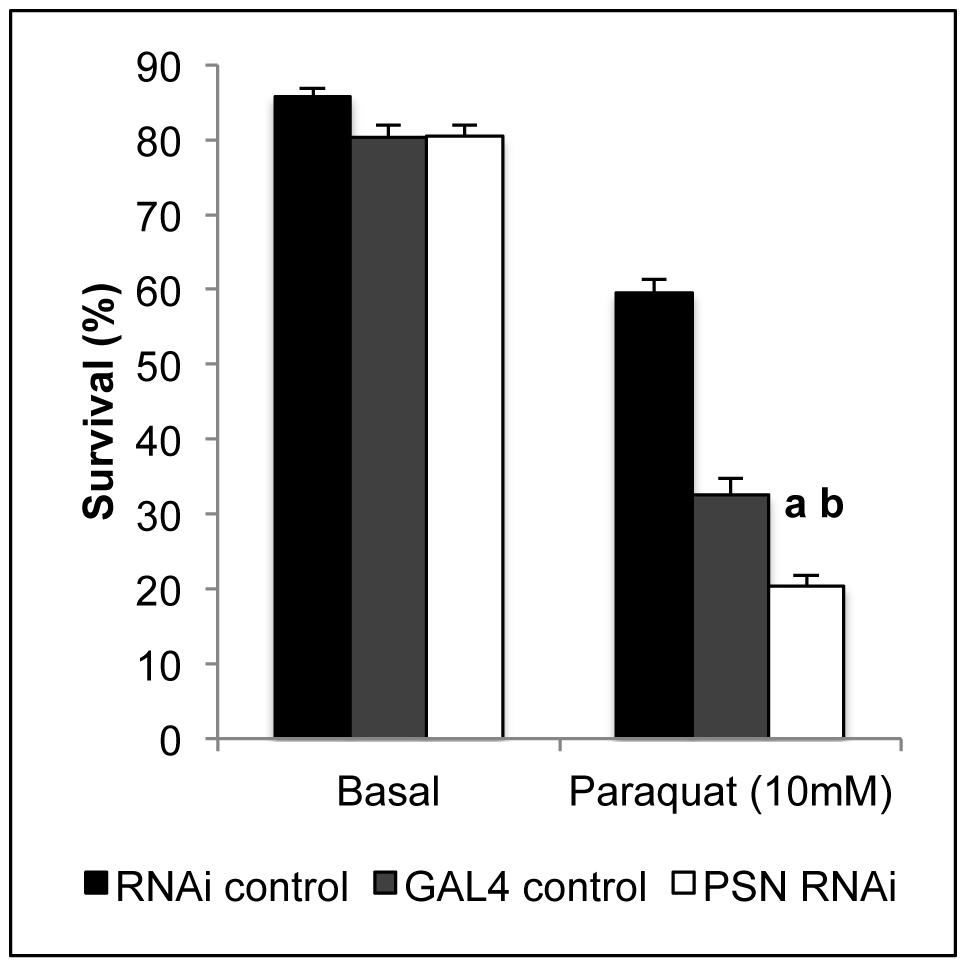
Oxidative stress tolerance in *Drosophila*. Paraquat, a potent inducer of superoxide, was used to assess oxidative stress tolerance. Survival to adulthood was assessed on basal media and media supplemented with 10 mM paraquat for control (RNAi control, GAL4 control) and midgut *PSN* knockdown (PSN RNAi) *Drosophila*. Values are percentage survival with S.E.M. from fifteen replicates of 50 *Drosophila*. *PSN* knockdown *Drosophila* were more susceptible to paraquat-supplemented media, indicative of greater sensitivity to superoxide. ^a^Significant difference from RNAi control, ^b^significant difference from GAL4 control, as determined by two-way ANOVA with Tukey’s post-hoc test (P<0.001).

We next sought to determine whether the reduced dietary copper uptake associated with midgut *PSN* knockdown had any effect on activity of the cupro-enzyme SOD1 (Figure 5). Relative to each control strain, SOD activity was significantly reduced in midgut *PSN* knockdown larvae (Figure 5A). This effect was not due to reduced SOD1 protein levels as western immunoblotting demonstrated comparable levels of SOD1 monomer and dimer in each strain (Figure 5B). The reduction in SOD activity is consistent with a functional copper deficiency associated with midgut *PSN* knockdown. Given SOD is an antioxidant that functions in the dismutation of superoxide into hydrogen peroxide, we sought to determine whether midgut *PSN* knockdown *Drosophila* had any effect on tolerance to oxidative stress. *Drosophila* were reared on basal media or media supplemented with paraquat, a potent inducer of superoxide radicals, and survival to adulthood was measured (Figure 6). Midgut *PSN* knockdown significantly reduced survival on paraquat-supplemented media, compared to each control strain. This result is indicative of greater sensitivity to superoxide and is consistent with reduced SOD activity (Figure 5A). These results support the model whereby reduced *PSN* levels inhibit dietary copper uptake and bioavailability in *Drosophila*.

## Discussion

In addition to well characterized roles in Notch signalling and APP processing, presenilins have been implicated in a variety of cellular processes including: protein trafficking [20], calcium homeostasis [21], lysosomal protein degradation [22] and most recently copper and zinc homeostasis [4]. We now propose a conserved role for presenilin in regulating copper uptake in *Drosophila*.

Ubiquitous RNAi-mediated knockdown of *PSN* in *Drosophila* was lethal, presumably due to Notch-dependent developmental defects. However, viable flies were obtained with knockdown of *PSN* in specific tissues of the digestive system including the midgut, Malpighian tubules and fat body. The Malpighian tubules are analogous to the mammalian kidney and the fat body shares functional similarity with the liver and adipose tissue [23], [24]. Li and colleagues recently reported that heart-specific *PSN* knockdown did not cause lethality and that these flies displayed cardiac defects, an important finding given the identification of *presenilin* gene mutations in patients with dilated cardiomyopathy [25], [26]. Investigation of other tissue-specific effects in *Drosophila* may elucidate additional presenilin functions of relevance to human health.

Midgut-specific knockdown of *PSN* was associated with increased copper tolerance (Figure 1) and several lines of evidence suggest this is likely to be due to reduced uptake of dietary copper into the midgut. Firstly, *PSN* knockdown was able to overcome the copper-sensitivity induced by overexpression of *DmATP7* (Figure 2). Given DmATP7 functions to export copper across the basolateral membrane of the midgut into the circulation, this result indicates the affect of *PSN* knockdown occurs within the midgut. Secondly, total copper levels were reduced when *PSN* knockdown *Drosophila* were reared on either basal media or copper-supplemented media (Figure 3). Thirdly, midgut copper levels were also likely to be lower following *PSN* knockdown, given the reduced expression of a copper-responsive MTN-EYFP construct in the midgut of larvae raised on a copper-supplemented diet (Figure 4). These findings are also consistent with PS1 and PS2 being required for efficient copper uptake in mammalian systems [4].

While studies with mice found PS1 and PS2 were required for efficient uptake of copper and zinc [4], we did not find any evidence for midgut *PSN* knockdown effecting zinc homeostasis. Total zinc levels were not consistently different and survival on zinc-limited and zinc-supplemented media was not different to controls (Figures 1, 3). RNAi based studies cannot exclude the possibility that PSN may affect dietary zinc uptake, as midgut PSN levels were not completely abolished. PSN may also be required for zinc uptake in *Drosophila* tissues that were not examined. Iron, calcium, manganese and magnesium levels were also unchanged by midgut *PSN* knockdown (Figure S1), indicating the major effect of PSN on metal levels was restricted to copper.

Presenillins are involved in the trafficking and localisation of a range of metalloproteins including APP, transferrin and tyrosinase [27], [28]. Copper uptake in the midgut of *PSN* knockdown *Drosophila* could be reduced as a result of impaired localisation of Ctr1A and Ctr1B. We previously reported that reducing levels of Syntaxin 5, a SNARE protein involved in vesicle trafficking, reduced plasma membrane localized CTR1 and copper uptake in mammalian cells [29]. *Drosophila* heterozygous for a *Syntaxin 5* null allele also displayed reduced dietary copper uptake and increased copper tolerance that is remarkably similar to that seen with knockdown of *PSN* in the midgut (Figures 1 and 3). Studies are underway to determine whether PS1 and PS2 are required for the correct localisation of CTR1 and the ZIP proteins in mammalian systems, as this may explain the reduced copper and zinc uptake associated with reduced presenilin levels.

SOD activity was reduced by midgut *PSN* knockdown (Figure 5), which is consistent with a functional copper deficiency caused by reduced dietary uptake. However, SOD1 requires both copper and zinc to be active and we cannot exclude the possibility of reduced zinc bioavailability. In both mammalian and *Drosophila* systems the copper chaperone CCS is required for incorporation of copper into SOD1 [30], [31]. CCS levels were significantly reduced in PS-null MEFs, suggesting this may be a possible cause of the reduced SOD1 activity in these cells [4]. We were unable to test this hypothesis due to the lack of a CCS specific antibody for *Drosophila*. Q-PCR analysis of gene expression in third instar larvae found *CCS* transcript levels were unaltered following midgut *PSN* knockdown (data not shown). *PSN* knockdown was also associated with increased susceptibility to paraquat (Figure 6). Paraquat is a potent inducer of superoxide and this susceptibility is consistent with impaired SOD activity, as previously reported in *Drosophila* expressing a low-activity *SOD1* allele [32]. Increased oxidative stress is a hallmark of AD and reduced SOD1 activity has been reported in the brain of post-mortem AD patients as well as in a transgenic AD mouse model [33], [34]. Metal dyshomeostasis has been proposed as a central factor contributing to oxidative stress and the pathogenesis of AD [5]. Copper limitation reduces amyloid beta-mediated toxicity in *Drosophila* models of AD [35], [36]. *Amyloid Precursor Protein Like* (*APPL*), the *Drosophila* orthologue of *APP*, does not produce amyloid beta, therefore these models are created by transgenic expression of human amyloid beta peptide. Our results support a role for Presenilins in regulating copper homeostasis independently of amyloid beta, but cannot rule out the possibility that APPL is involved.

In conclusion, we propose that impaired presenilin activity inhibits dietary copper uptake in the gut of *Drosophila*, leading to a functional copper deficiency, reduced SOD activity and sensitivity to oxidative stress. This model is consistent with previous studies of presenilin in mammalian systems, and we hypothesise that presenilins have a conserved role in the regulation of dietary copper uptake.

## Supporting Information

Figure S1 **Tolerance to copper limitation in** ***Drosophila.*** Tolerance to copper limitation was assessed by survival to adulthood on basal media and media supplemented with the copper chelator BCS (0.5 mM) for control (RNAi control, GAL4 control) and midgut *PSN* knockdown (*PSN* RNAi) *Drosophila*. Values are percentage survival with S.E.M. from fifteen replicates of 50 *Drosophila*. Relative to basal media, BCS supplemented media did not significantly affect the survival of control or *PSN* knockdown *Drosophila*. When reared on BCS supplemented media, survival of *PSN* knockdown *Drosophila* was significantly lower than that of controls. ^a^Significant difference from RNAi control, ^b^significant difference from GAL4 control, as determined by one-way ANOVA with Tukey’s post-hoc test (P<0.05).
(JPG)

Figure S2 **Metal accumulation in** ***Drosophila.*** Iron (A), manganese (B), calcium (C) and magnesium (D) levels were measured by ICP-MS in control (RNAi control, GAL4 control) and midgut *PSN* knockdown (PSN RNAi) *Drosophila* pupae reared on basal media. Values are mean metal content per pupa with S.E.M. from five replicates of 20 pupae. ^a^Significant difference from GAL4 control as determined by one-way ANOVA with Tukey’s post-hoc test (P<0.01). *PSN* knockdown did not significantly affect iron, manganese or magnesium levels when compared to either the RNAi control or GAL4 control. *PSN* knockdown significantly affect calcium levels when compared to the GAL4 control, but not the RNAi control.
(JPG)
