# Supplementary figures and images for: Presenilin Promotes Dietary Copper Uptake
Source: PLoS One. 2013 May 7;8(5):e62811. doi: 10.1371/journal.pone.0062811 (PMC3646984; doi:10.1371/journal.pone.0062811)

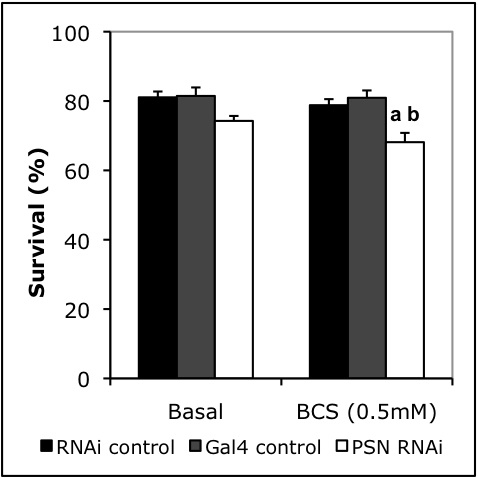

Supplement: Figure S1 — Tolerance to copper limitation in Drosophila. Tolerance to copper limitation was assessed by survival to adulthood on basal media and media supplemented with the copper chelator BCS (0.5 mM) for control (RNAi control, GAL4 control) and midgut PSN knockdown (PSN RNAi) Drosophila. Values are percentage survival with S.E.M. from fifteen replicates of 50 Drosophila. Relative to basal media, BCS supplemented media did not significantly affect the survival of control or PSN knockdown Drosophila. When reared on BCS supplemented media, survival of PSN knockdown Drosophila was significantly lower than that of controls. aSignificant difference from RNAi control, bsignificant difference from GAL4 control, as determined by one-way ANOVA with Tukey’s post-hoc test (P<0.05). (JPG) [file pone.0062811.s001.jpg]

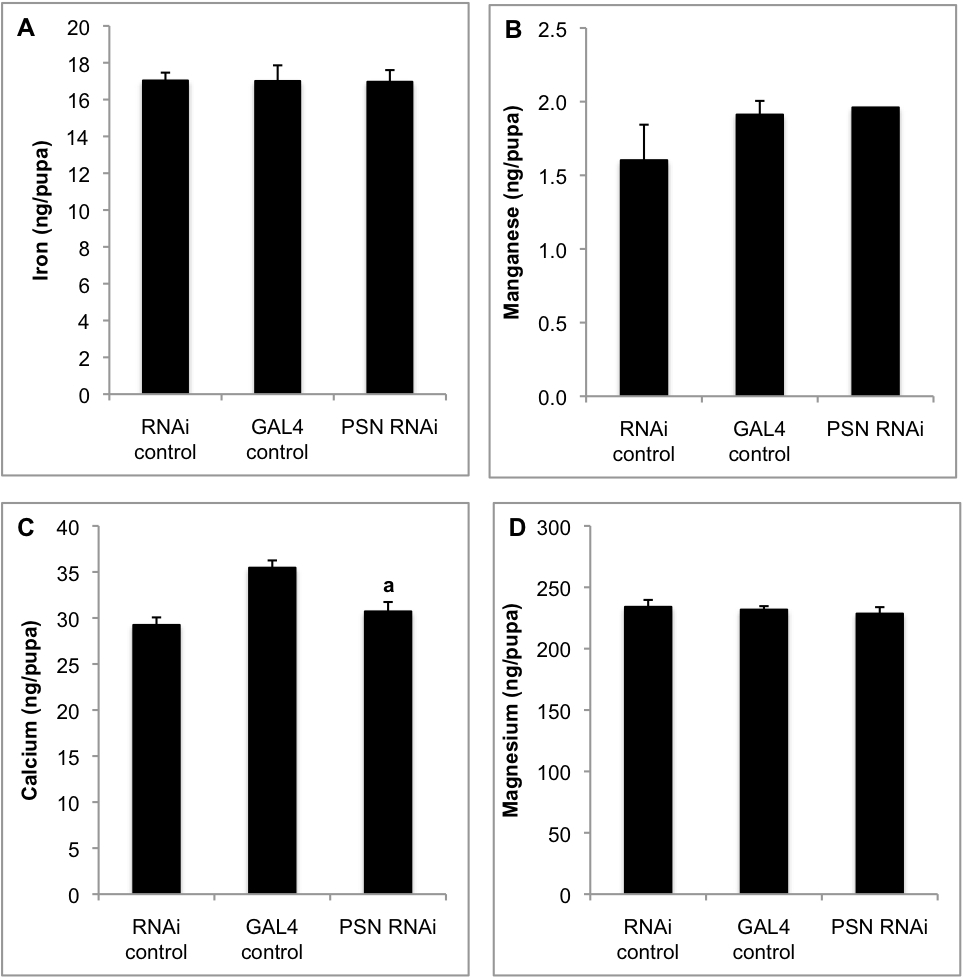

Supplement: Figure S2 — Metal accumulation in Drosophila. Iron (A), manganese (B), calcium (C) and magnesium (D) levels were measured by ICP-MS in control (RNAi control, GAL4 control) and midgut PSN knockdown (PSN RNAi) Drosophila pupae reared on basal media. Values are mean metal content per pupa with S.E.M. from five replicates of 20 pupae. aSignificant difference from GAL4 control as determined by one-way ANOVA with Tukey’s post-hoc test (P<0.01). PSN knockdown did not significantly affect iron, manganese or magnesium levels when compared to either the RNAi control or GAL4 control. PSN knockdown significantly affect calcium levels when compared to the GAL4 control, but not the RNAi control. (JPG) [file pone.0062811.s002.jpg]
